# Stimulus Feature-Specific Information Flow Along the Columnar Cortical Microcircuit Revealed by Multivariate Laminar Spiking Analysis

**DOI:** 10.3389/fnsys.2020.600601

**Published:** 2020-11-30

**Authors:** David A. Tovar, Jacob A. Westerberg, Michele A. Cox, Kacie Dougherty, Thomas A. Carlson, Mark T. Wallace, Alexander Maier

**Affiliations:** ^1^Neuroscience Program, Vanderbilt University, Nashville, TN, United States; ^2^School of Medicine, Vanderbilt University, Nashville, TN, United States; ^3^Department of Psychology, Vanderbilt University, Nashville, TN, United States; ^4^Center for Integrative and Cognitive Neuroscience, Vanderbilt University, Nashville, TN, United States; ^5^Vanderbilt Vision Research Center, Vanderbilt University, Nashville, TN, United States; ^6^Center for Visual Science, University of Rochester, Rochester, NY, United States; ^7^Princeton Neuroscience Institute, Princeton University, Princeton, NJ, United States; ^8^School of Psychology, University of Sydney, Sydney, NSW, Australia; ^9^Department of Hearing and Speech Sciences, Vanderbilt University, Nashville, TN, United States; ^10^Department of Psychiatry, Vanderbilt University, Nashville, TN, United States; ^11^Kennedy Center for Research on Human Development, Vanderbilt University, Nashville, TN, United States

**Keywords:** cortical layers, cortical microcircuit, macaque, rhesus, machine learning, vision, visual cortex (V1)

## Abstract

Most of the mammalian neocortex is comprised of a highly similar anatomical structure, consisting of a granular cell layer between superficial and deep layers. Even so, different cortical areas process different information. Taken together, this suggests that cortex features a canonical functional microcircuit that supports region-specific information processing. For example, the primate primary visual cortex (V1) combines the two eyes' signals, extracts stimulus orientation, and integrates contextual information such as visual stimulation history. These processes co-occur during the same laminar stimulation sequence that is triggered by the onset of visual stimuli. Yet, we still know little regarding the laminar processing differences that are specific to each of these types of stimulus information. Univariate analysis techniques have provided great insight by examining one electrode at a time or by studying average responses across multiple electrodes. Here we focus on multivariate statistics to examine response patterns across electrodes instead. Specifically, we applied multivariate pattern analysis (MVPA) to linear multielectrode array recordings of laminar spiking responses to decode information regarding the eye-of-origin, stimulus orientation, and stimulus repetition. MVPA differs from conventional univariate approaches in that it examines patterns of neural activity across simultaneously recorded electrode sites. We were curious whether this added dimensionality could reveal neural processes on the population level that are challenging to detect when measuring brain activity without the context of neighboring recording sites. We found that eye-of-origin information was decodable for the entire duration of stimulus presentation, but diminished in the deepest layers of V1. Conversely, orientation information was transient and equally pronounced along all layers. More importantly, using time-resolved MVPA, we were able to evaluate laminar response properties beyond those yielded by univariate analyses. Specifically, we performed a time generalization analysis by training a classifier at one point of the neural response and testing its performance throughout the remaining period of stimulation. Using this technique, we demonstrate repeating (reverberating) patterns of neural activity that have not previously been observed using standard univariate approaches.

## Introduction

Certain anatomical motifs are repeated across disparate brain areas with wide-ranging functions. The mammalian neocortex is one such example as it predominantly features the same laminar structure. A popular model for cortical function resting upon this stereotypical structure is the canonical cortical microcircuit (CCM: Douglas et al., 1989; Douglas and Martin, 1991; Bastos et al., 2012). The CCM gives rise to a series of distinct, yet overlapping, activation steps that are spatially segregated between the superficial (supragranular), deep (infragranular), and middle (granular) layers of cortex (Rockland and Pandya, 1979; Rockland and Virga, 1989; Callaway, 1998; Binzegger et al., 2004; Douglas and Martin, 2004). According to this model, ascending (feedforward) signals from parts of the brain that are closer to the sensory periphery terminate in the middle layers of cortical areas while descending (feedback) signals from downstream areas target the layers above and below (Rockland and Pandya, 1979; Rockland and Virga, 1989; Felleman and Van Essen, 1991, but see Self et al., 2013).

Since the CCM applies virtually ubiquitously across neocortex, an improved understanding of the laminar cortical processing chain is bound to translate into an improved understanding of cortical processing more generally (Hubel and Wiesel, 1977; Douglas et al., 1989; Felleman and Van Essen, 1991; Douglas and Martin, 2004; Bastos et al., 2012). Our knowledge of laminar neural activity in primates has grown greatly over the last decade thanks to the prevalence of linear electrode arrays (Schroeder et al., 1998; Xing et al., 2009, 2012; Burns et al., 2010; Buffalo et al., 2011; Kajikawa and Schroeder, 2011; Maier et al., 2011, 2014; Hansen et al., 2012; Spaak et al., 2012; Smith et al., 2013; Bastos et al., 2014, 2018; Van Kerkoerle et al., 2014; Nandy et al., 2017; Cox et al., 2019a,b; Westerberg et al., 2019; Dougherty et al., 2019a; Gieselmann and Thiele, 2020). Yet, our knowledge about laminar neuronal activation remains limited (e.g., Mignard and Malpeli, 1991). Recent studies demonstrated that—matching predictions by the CCM—there are two distinct sequences of laminar activation for feedforward and feedback activation, respectively (Maier, 2013; Van Kerkoerle et al., 2014, 2017; Cox et al., 2019a). Much less is known about the different types of feedforward processes that occur along cortical layers. Specifically, we still know little about how one and the same feedforward sweep of neural activation across cortical layers entails multiple streams of stimulus-specific information that manifest differently across space and time.

Our knowledge regarding laminar cortical processing is bound to rapidly increase since there have been notable advances in microelectrode technology. Specifically, the increase in simultaneously placed electrodes and the associated increase dimensionality of laminar neurophysiological data obtained by second generation laminar arrays is rapidly approaching those of other techniques such as fMRI (Jun et al., 2017; Steinmetz et al., 2018; Musk and Neuralink, 2019). Yet, laminar recordings are usually analyzed using the same univariate techniques that have been established for single electrodes, rather than utilizing the additional, contextual information provided by neighboring electrode contacts in a multivariate fashion.

There are several statistical approaches that quantify information distributed across neighboring measurements in the brain, directly capturing neuronal interactions on the population level. Specifically, machine-learning based multivariate pattern classification analysis (MVPA) has proven fruitful in systems neuroscience (Haxby et al., 2001; Kriegeskorte and Bandettini, 2007; Kriegeskorte et al., 2008; Kriegeskorte and Kreiman, 2012; Rutishauser et al., 2018). More recently, time-resolved MVPA has emerged as a powerful technique to study the time courses with which information processing occurs across the brain (Carlson et al., 2013; Cichy and Pantazis, 2017; Tovar et al., 2020). While time-resolved MVPA has been applied to multielectrode recordings (Goddard et al., 2017), to date no study to our knowledge probed whether this technique can reveal aspects of laminar cortical activation that are opaque to univariate analyses. For instance, through time generalization, which is achieved by training a classifier at a specific time point—such as early in the neuronal response to a stimulus—then testing it throughout the remainder of the response, one can search for repeating patterns of neural activity across electrodes that might be invisible when analyzing single channels in isolation.

Here we use time-resolved MVPA to analyze the pattern of spiking activity across 24 and 32 channel (first generation) linear multielectrode array recordings in primate primary visual cortex (V1). Instead of relying on the average response across all electrode channels or only examining one channel at a time, MVPA uses patterns of activity across neighboring channels to classify neuronal responses. We use both time-resolved MVPA and an MVPA-based “searchlight” analysis commonly used for neuroimaging data to map how information regarding stimulus orientation, eye-of-origin, and stimulus history differentially flows within the laminar activation sequence of V1. We found that MVPA can be utilized effectively despite the relatively low channel counts of first generation laminar linear arrays. We then explored time-generalization, as this analysis provides insight that cannot be gained from more conventional, univariate approaches that are blind to patterns of activity that span multiple electrodes. This analysis revealed repeating patterns in neuronal activity that entailed information about whether a stimulus had previously been shown or not, which we had not observed in a prior study that had relied on univariate analyses exclusively (Westerberg et al., 2019). We discuss these findings and their implications for the advent of massively increased channel counts for linear multielectrode arrays that are rapidly gaining prominence (Jun et al., 2017; Steinmetz et al., 2018; Musk and Neuralink, 2019).

## Materials and Methods

### Animal Care and Surgical Procedures

Data were collected from two macaque monkeys [*Macaca radiata*, one female (designated Monkey 1) and one male (designated Monkey 2)]. All procedures were in compliance with regulations set forth by the Association for the Assessment and Accreditation of Laboratory Animal Care (AALAC), approved by the Vanderbilt University Institutional Animal Care and Use Committee, and followed National Institutes of Health guidelines. A detailed description of the surgical procedures can be found in previous publications (Westerberg et al., 2019, 2020a,b). Briefly, in a series of surgeries, each monkey was implanted with a custom MRI-compatible headholder and recording chamber over perifoveal V1 concurrent with a craniotomy.

### Behavioral Paradigm

**Figure 1:**
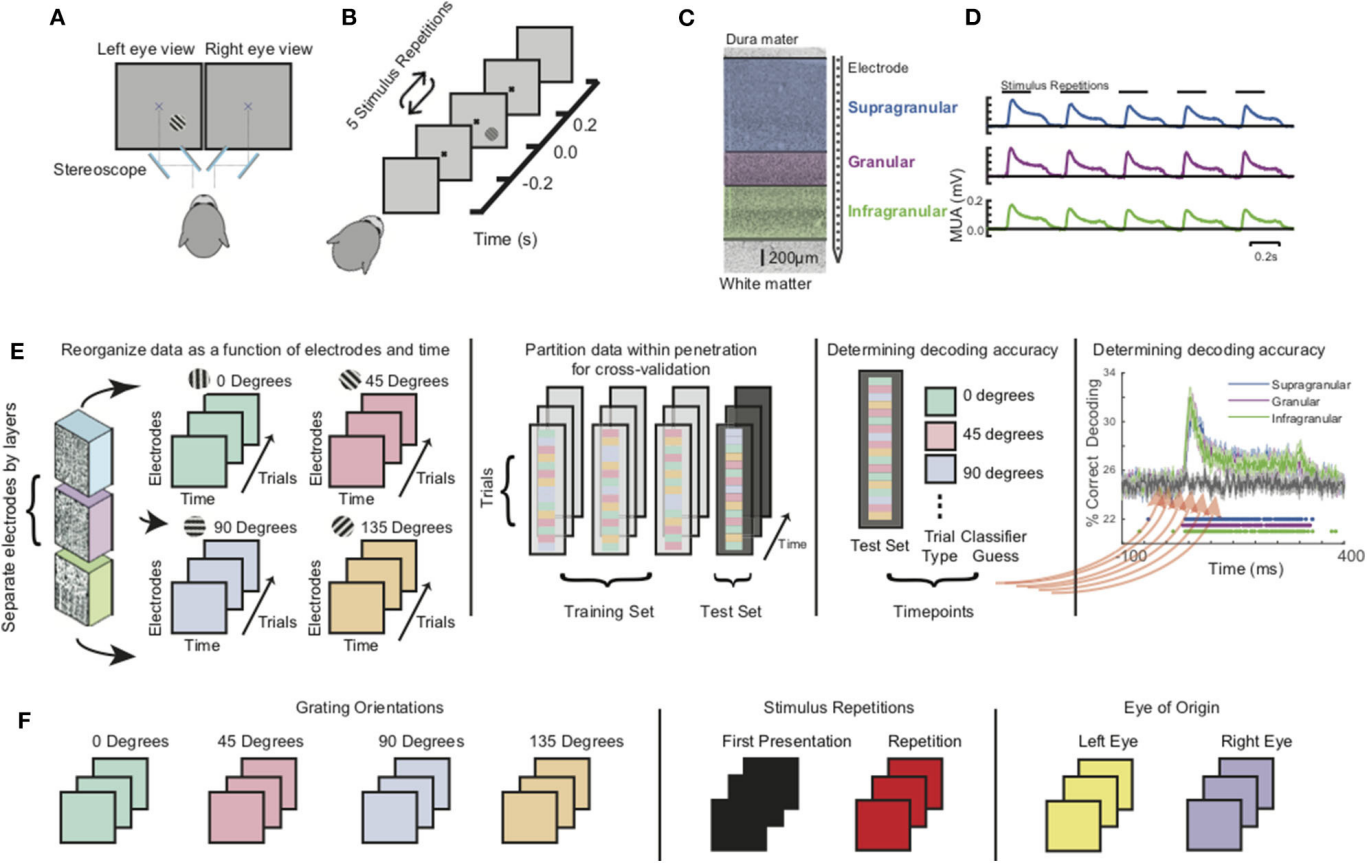
Experimental setup, paradigm, preprocessing, and analysis. **(A)** Monkeys were positioned in front of a monitor and tasked to passively fixate a central dot through a custom mirror stereoscope. **(B)** Monkeys were shown a series of five grating stimuli of randomly varying orientations and ocular configuration with all other parameters were held constant. **(C)** Linear multicontact array recording laminar neuronal responses at 100 micron spatial resolution spanning through visual cortex. **(D)** Grand average multiunit spiking responses (MUA) to the stimulus sequence for all three main laminar compartments (both animals, all sessions). **(E)** Schematic of multivariate pattern analysis (MVPA). Population spiking responses (MUA) from each laminar compartment were reorganized as a function of electrode contact and time. A classifier was trained at each timepoint using linear discriminant analysis and 4-fold cross validation. **(F)** Decoding analysis was separately performed for grating orientations, stimulus history (initial stimulus vs. repetitions), and eye-of-origin.

In each recording session, monkeys viewed a 20” CRT monitor (Diamond Plus 2020u, Mitsubishi Electric Inc.) operating at 60 or 85 Hz. Monkeys passively fixated within a one-degree radius around a central fixation dot and viewed stimuli through a custom mirror stereoscope so that stimuli could be viewed monocularly or binocularly (Figure 1A). To eliminate potential response differences due to binocular disparity, prior to the main tasks, a mirror calibration task was performed. In this task, monkeys shifted gaze to a series of stimuli positioned across the visual display and held fixation at each position to receive fluid reward. Each stimulus was presented to only one eye at a time. This resulted in two maps of fixation positions, one for the set of stimuli presented to each eye. The stereoscope was then adjusted if differences were observed in those maps (e.g., the maps were not completely overlapping). Stimuli were generated using MonkeyLogic (Asaad et al., 2013; Hwang et al., 2019) via MATLAB (R2012, R2014a, The Mathworks, Inc.) running on a computer using a Nvidia graphics card. Following 300 ms of fixation, monkeys viewed five sequentially presented stimuli for 200 ms each, with a 200 ms inter-stimulus interval (ISI). If fixation was maintained throughout the five presentations, the monkey was rewarded with juice and relieved of the fixation constraint for an inter-trial interval (ITI). If the monkey broke fixation during trial performance, the presentation was eliminated from analysis and the monkey experienced a short timeout (1–5 s) before starting the next trial. Each stimulus in the presentation sequence was a sinusoidal bar grating of equivalent size, spatial frequency, and phase, with variable orientation and eye-of-origin (Figure 1B). For each recording session, the stimuli were optimized for the measured neural activity evaluated by listening to the multi-unit activity (MUA) during exposure to a wide variety of stimuli. We selected stimulus parameters that evoked the greatest neural response. For a more detailed description of the paradigm, as well as further information on stimulus optimization and receptive field mapping (Supplementary Figure 1), see previous publications (Cox et al., 2013, 2019a,b; Dougherty et al., 2019a; Westerberg et al., 2019).

### Neurophysiological Procedure

All data used in this paper are available upon request from the communicating author, pending approval by Vanderbilt University. During task performance, broadband (0.5 Hz−12.207 kHz) intracranial voltage measurements were taken at a sampling rate of 30 kHz and amplified, filtered, digitized using a 128-channel Cerebus™ Neural Signal Processing System (NSP, Blackrock Microsystems LLC). Neuronal data was downsampled offline to 1 kHz, following low-pass filtering with an anti-aliasing filter. Gaze position was recorded at 1 kHz (NIDAQ PCI-6229, National Instruments) using an infrared light sensitive camera and commercially available eye tracking software (Eye Link II, SR Research Ltd.; iView, SensoMotoric Instruments). Recordings took place inside an electromagnetic radio frequency-shielded booth and were performed using one or two acute laminar multielectrode arrays with 24 or 32 contacts with 0.1 mm electrode spacing and impedances ranging between 0.2 and 0.8 megaohms at 1 kHz (U-Probe, Plexon, Inc.; Vector Array™, NeuroNexus). Electrodes were connected to the NSP using analog headstages. In each recording, the electrode array(s) were introduced into dorsal V1 through the intact dura mater using a chamber-mounted microdrive (custom modification of a Narishige International Inc. Micromanipulator) and adjusted such that the majority of recording contacts spanned the cortical sheet. This procedure was repeated across the 61 experimental sessions (*n* = 13 for monkey I34).

### Receptive Field Mapping

Since achieving single-unit isolation on every channel is difficult, we instead opted to estimate the local population spiking response by quantifying the time-varying activity in the spiking frequency range (multi-unit activity, MUA) as we wanted to ensure overlapping receptive fields along the cortical depth. Verifying overlapping receptive fields provides confidence that the activity we are recording across columns originates from the same cortical location rather than spanning adjacent columns (i.e., that the electrode penetration was orthogonal to cortex). Monkeys performed a visual fixation task where a visual stimulus was presented repeatedly in the contralateral visual hemifield – relative to the position of the electrode array. Up to five stimuli were presented on each trial for 200 ms with a 200 ms interstimulus interval. Stimulus size and positioned varied between recording sessions, but each session usually consisted of a “coarse” receptive field mapping task followed by a more focused version once an estimation for the exact position was found. We mapped receptive fields using a reverse-correlation technique (Supplementary Figure 1) which resulted in 3-dimensional receptive field matrices where 2 dimensions corresponded to visual space and the third, response magnitude (Cox et al., 2013). Only sessions where the receptive field matrices were overlapping along cortical depth were included for further analysis. Additionally, this procedure determined the position where the stimulus was positioned to stimulate the column receptive field for the main task (see section Behavioral Paradigm).

### Laminar Alignment

Current source density (CSD) in response to brief visual stimulation was used to find the boundary between the granular and infragranular compartments of V1 as per previously documented methods (Schroeder et al., 1998; Maier et al., 2010; Maier, 2013; Ninomiya et al., 2015; Cox et al., 2019a,b; Dougherty et al., 2019a; Westerberg et al., 2019). Only sessions that were found to be perpendicular to the cortical surface were included in analysis (see section Receptive Field Mapping). Additional neurophysiological criteria were used, such as well-defined patterns of LFP power spectral density (Van Kerkoerle et al., 2014; Bastos et al., 2018; Westerberg et al., 2019), signal correlations between LFP recorded on differing channels (Westerberg et al., 2019), and latency (Self et al., 2013) of stimulus-evoked MUA. The granular to supragranular boundary was set to 0.5 mm above the granular to infragranular boundary (Figure 1C). Supplementary Figure 2 demonstrates the reliability of these functional markers following alignment of all sessions. Both extracranial to intracranial and gray matter to white matter boundaries were determined by finding the pair of recording electrodes where no multiunit response to visual stimuli was observed on one channel and a significant response was observed on the other (Cox et al., 2019b; Westerberg et al., 2019). Recording channels positioned between these pairs all showed significant responses. That is, we found no instances of a lack of response on a channel determined to be within the gray matter. The L2/3–L4 boundary was set to 0.5 mm above the L4–L5 boundary as we do not have a reliable functional marker and that distance is consistent with histological studies of V1 laminar structure (see Cox et al., 2019b; Westerberg et al., 2019 for details).

### Data Preprocessing

All contiguous recording channels found to be within the gray matter were taken and multiunit signals were computed. Channels in the gray matter were found by determining first whether a visual response could be evoked on the channel and second, whether a receptive field was present for the multiunit and/or LFP activity through a previously described receptive field mapping paradigm (Westerberg et al., 2019). If the channel was found to be in the gray matter, the broadband neural signal recorded at that channel was then band-pass filtered between 500 and 5,000 Hz, rectified, and low-pass filtered at 200 Hz using Butterworth filters (Self et al., 2013; Shapcott et al., 2016; Westerberg et al., 2020a). These derived neural signals, with no further filtering of the multiunit activity, were then used in performing both the univariate and multivariate analyses (Figure 1D).

### Multivariate Pattern Analysis

To track how sensory information from different stimulus features are processed within this laminar microcircuit, we applied multivariate pattern analysis (MVPA) using CoSMoMVPA (Oosterhof et al., 2016) to the MUA of each of the three laminar compartments (Figure 1E, left-most panel). To do so, we assembled two-dimensional neuronal response matrices (NRMs) that contained the millisecond-by-millisecond population spiking response at each electrode channel as a function of trials. Each row/electrode in the NRM can be thought of as a separate axis forming a multidimensional space whose dimensionality is determined by the number of electrodes. Each stimulus presentation will elicit a different response across each of the dimensions. The specific stimulus features we tested comprised of grating orientation, the eye that the stimuli were presented to (eye-of-origin) and the relative position of each stimulus within the stimulation sequence (Figure 1F). We next randomly divided trials within sessions to perform a 4-fold cross-validation procedure. In this procedure, 3/4 of the data is used to train an MVPA classifier (Figure 1E, second-to-left panel). The remaining 1/4 of the NRMs are used to determine classifier performance. To classify a given stimulus feature, a different hyperplane or set of hyperplanes (as is the case with the orientation where we have four orientations) is used to distinguish stimulus feature on a trial by trial basis. The decoding accuracy is the number of trials over the total number of trials that classifier is able to correctly identify for each session. We performed this computation separately within each recording session on a millisecond-by-millisecond basis, evaluating the accuracy of classifier performance as a function of time (Figure 1E, second-to-rightmost panel). The resulting time courses of decoding accuracy for each laminar compartment were then pooled together and compared to a randomized trial shuffle control to determine statistical significance (Figure 1E, rightmost panel). To correct for multiple comparisons, we used the false discovery rate (FDR) adjusted *p*-values with α = 0.01. For each of the decoding distinctions, the subsets were balanced, such that both training subsets and testing subsets contained the same number of trials for each stimulus category.

For orientation decoding, all recording sessions were used for analysis. However, some recording sessions included orientation presentations that were not shown in other recording sessions (i.e., 22.5° in one recording session and 30° in another sessions). Therefore, orientation presentations were binned into four categories: 0–44°, 45–89°, 90–134°, and 135–179°. For trial repetition decoding, the five stimuli presentations for a given trial were grouped as either the first presentation or as a repetition. To have an equal number of first presentations and repetitions, we randomly subsampled from the repetitions to match the number of first presentations.

For each stimulus feature, we also performed a time generalization analysis (Carlson et al., 2011; King and Dehaene, 2014) which uses a similar decoding procedure described, with one notable exception — the classifier is trained on the information at one time point for each stimulus feature and the model is subsequently tested on all timepoints. This procedure is repeated across all timepoints resulting in a 2D “time generalization matrix” that plots training time against decoding time to gain insight into how information at specific timepoints evolve throughout the time course. Lastly, to determine the effects of repeated stimuli presentations on orientation and eye of origin decoding, we further divided the repetition subset of data into balanced eye of origin subsets and balanced orientation subsets. We then again performed a 4-fold classification using a linear discriminant analysis classifier.

## Results

### Stimulus Feature-Specific Information Within Neural Activation of the CCM

**Figure 2:**
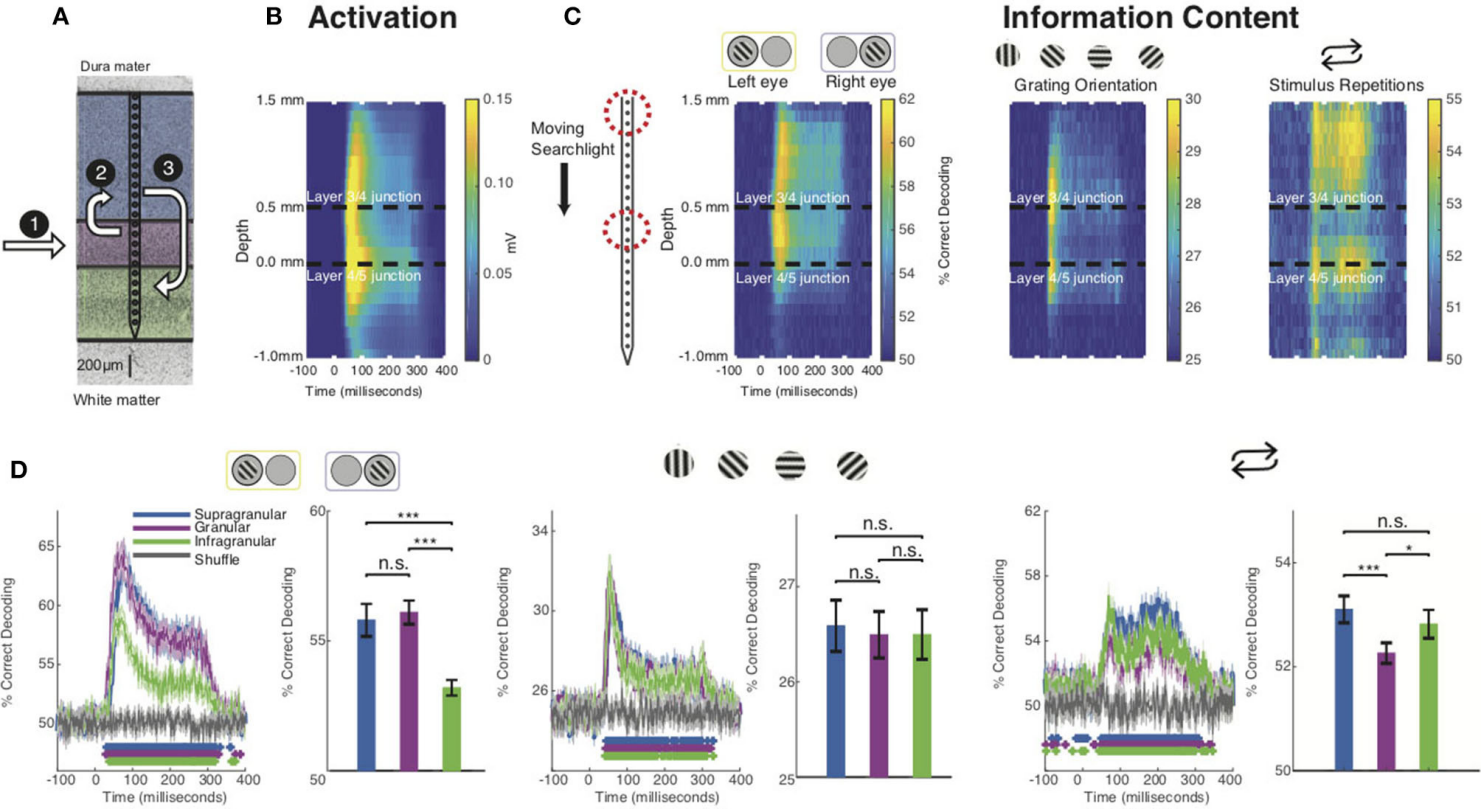
Stimulus feature-specific information within neural activation of the CCM. **(A)** Canonical microcircuit model (CCM) of neural activation in V1. Feedforward activation initially excites the middle layers before reaching upper and lower layers of cortex. **(B)** Grand average laminar MUA profile to all stimulus presentations along the depth of the electrode (all sessions, both monkeys). **(C)** Decoding performance using a “moving searchlight” along the electrode array for eye of origin (leftmost panel), grating orientation (middle panel), and stimulus repetition (rightmost panel). **(D)** Time series of MVPA decoding for eye of origin (leftmost panel), grating orientation (middle panel), and stimulus repetitions (rightmost panel). Graphs show decoding accuracy as a function of time and laminar compartment, together with a randomized shuffled control as a baseline. Significance is indicated with colored asterisks above the abscissa using Wilcoxon signed-rank test, FDR corrected, *q* < 0.01. Bar plots to the right indicate time-averaged statistics of the data with Wilcoxon signed-rank test *P* values (*p < 0.05, **p < 0.01, ***p < 0.001) above the plots.

Before investigating each stimulus feature in isolation, we evaluated whether the grand average spiking response to our stimuli matched predictions from the CCM (Figure 2A). To do so, we spatially aligned the spiking data from each recording session to the layer 4C/5 boundary. Using these aligned datasets, we computed the grand average spiking response to all stimuli as a function of cortical depth and time (Figure 2B). The resulting laminar profile of activation was consistent with both the expectations set by the CCM and previous studies of laminar visual activation in that layer 4C activity preceded that of the other layers (Mitzdorf, 1985; Schroeder et al., 1998; Maier et al., 2010; Spaak et al., 2012; Van Kerkoerle et al., 2014). Interestingly, however, both the supragranular and infragranular layers responded virtually simultaneously, which might either be explained by (i) V1's idiosyncratic laminar connections [i.e., there are also, less pronounced, geniculate projections outside layer 4C (Callaway, 1998)], (ii) limitations of the CCM model itself (e.g., Godlove et al., 2014; Ninomiya et al., 2015), or both. This pattern of sensory activation occurs regardless of stimulus feature, raising the question of how stimulus-specific information is extracted within this activation sequence. To answer this question, we applied MVPA using a “moving searchlight” analysis (Etzel et al., 2013). Specifically, we limited both our training and test data sets to three neighboring electrode channels, performed MVPA over time, and then repeated the process after moving this “searchlight” 0.1 mm deeper along the electrode array. In this analysis a classifier is trained and tested for each timepoint of the response, in 1 ms increments (Figure 2C). No spatial or temporal smoothing were added.

We first focused on the eye-of-origin for each stimulus presentation. While V1 harbors both neurons that respond to one or both eyes, most of the neurons that respond to one eye only (monocular neurons) are located in the middle, granular layers (Hubel and Wiesel, 1977; Dougherty et al., 2019a). This finding is consistent with neuroanatomy, as the granular layers receive the bulk of (monocular, eye-specific) inputs from the lateral geniculate nucleus of the thalamus (LGN) that connects eye and cortex (Casagrande and Boyd, 1996). A long-standing hypothesis is that the eye-specific inputs in the middle layers are merged to a combined (binocular) response in the layers above, even though most V1 neurons maintain preference for one eye over the other (Hubel and Wiesel, 1972; Ohzawa and Freeman, 1986; Prince et al., 2002; Read and Cumming, 2004). Neurons in the uppermost layers of V1 project to neurons in V1's lower layers, so if the upper layers form a combined binocular signal, this signal should be present in the lower layers as well (Hubel and Wiesel, 1972; Cox et al., 2019b; Dougherty et al., 2019a). However, based on several other pieces of empirical evidence, an alternative hypothesis postulates that the two eyes' signals are interacting at or before LGN responses arrive in the middle layers of V1 (see Dougherty et al., 2019b for review).

Using MVPA, we found information regarding eye-of-origin initially followed the CCM profile of general activation, with neurons reliably indicating whether a stimulus was shown to left or right eye in the middle layers, followed by the upper layers of V1. This eye-specific information largely diminished once neuronal activation reached the lower layers of V1 (Figure 2C, left panel). These timing differences can clearly be seen for a layer-specific MVPA using all electrode channels within the middle, upper and lower layers of V1, respectively (Figure 2D). We utilized this analysis to perform several statistical comparisons. First, we compared decoding performance on a millisecond-by-millisecond basis against a randomized trial shuffle control. Second, we compared decoding across laminar compartments. Decoding of eye-of-origin first emerged in the middle layers (29 ms), followed by the upper (40 ms) and lower layers (40 ms). Decoding which eye the stimuli were shown to was comparable between middle and upper layers but significantly reduced in the lower layers, suggesting that eye-specific information is largely preserved when granular neurons project to neurons in the layers above. However, decoding of eye-of-origin is relatively poor in the lower layers of V1, suggesting that, at least on the multiunit-level, there is significant binocular convergence after activation reaches the upper layers of cortex. This finding demonstrates that eye-of-origin is more robustly represented in supragranular compared to infragranular layers.

Next, we computed the laminar evolution of stimulus orientation information. A common notion regarding the functional layout of V1 states that orientation selectivity (tuning) is less pronounced in the middle layers of V1 (Hubel and Wiesel, 1972, 1977; Ringach et al., 2002). Several authors have since challenged this idea, arguing that V1 already receives orientation-biased inputs (Daniels et al., 1977; Vidyasagar and Urbas, 1982; Leventhal and Schall, 1983; Smith et al., 1990; Pugh et al., 2000; Xu et al., 2002). We thus wondered what the laminar profile of MVPA-based decoding of stimulus orientation across V1 layers might be.

We binned our grating stimuli into four groups (0°, 45°, 90°, and 135°, respectively) and trained a classifier to discriminate between them (Figure 2C). Interestingly, we found that information regarding stimulus orientation was more transient than information regarding of eye-of-origin. Moreover, the laminar profile was strikingly different: the center of the granular layers discriminated relatively poorly between gratings of varying orientation, and neurons in the layers above and below did so without any significant temporal delay. Closer inspection of the layer-resolved decoding (Figure 2D), collapsed across time, revealed that there was no significant difference between any of the laminar compartments (bar plots). These results seem to suggest that stimulus orientation information is extracted almost uniformly across V1 layers. However, visual inspection reveals clear differentiation within the middle layers, which is lost when collapsing this layer into a single measure. This heterogeneous pattern within the granular layers might at least be partially explained by the fact that the middle layers host several sublayers that each receive separate inputs from the LGN (Casagrande and Boyd, 1996), although it is not immediately clear how the granular sublayers relate to the specific pattern we found.

Given that V1 is known to modulate its responses depending on contextual cues, such as the behavioral state of the animal or stimulus history (Van Kerkoerle et al., 2014; Cox et al., 2019a; Westerberg et al., 2019), we next examined how stimulus history affects the laminar flow of stimulus-specific information. To do so, we first studied the laminar flow of information of whether a stimulus was novel or preceded by another stimulus in the stimulation sequence. We found that this information regarding stimulus history yielded yet another pattern of laminar information flow (Figure 2C). We found that the bulk of information regarding stimulus history resided outside the granular input layers. This finding was also apparent in layer-specific MVPA (Figure 2D). These results are in line with earlier work showing that V1 granular layers are least affected by the adaptive effects of repeated visual stimulation (Westerberg et al., 2019).

### Quantifying Differences Between Spatiotemporal Searchlight Maps

**Figure 3:**
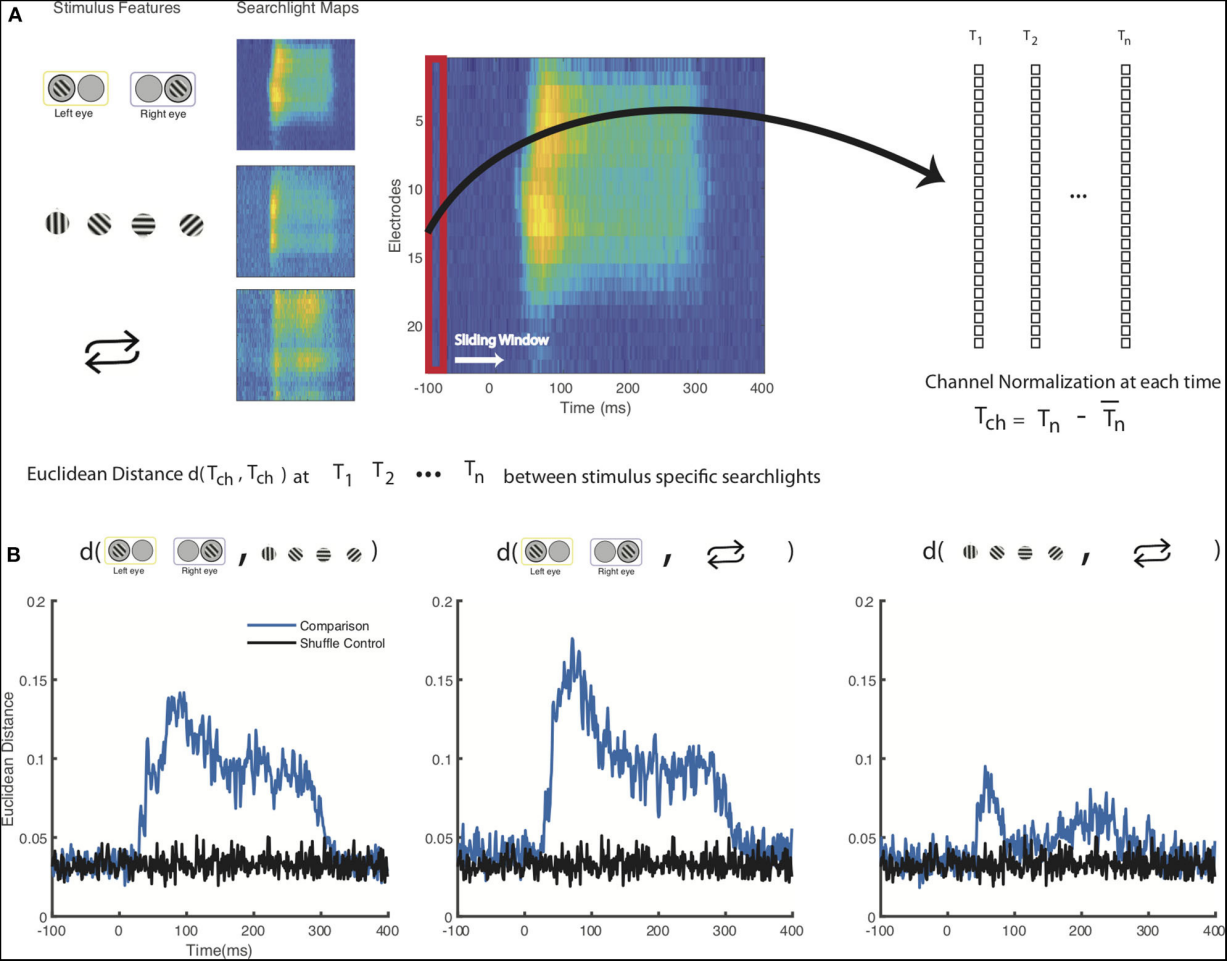
Statistical comparison of columnar flow of stimulus feature-specific information. **(A)** Schematic for comparison between stimulus-feature specific searchlight analyses. Decoding results from the searchlight analyses for each of the stimulus features, normalized across all the channels for each individual timepoint from 100 ms prior to stimulus presentation to 400 ms after stimulus presentation **(B)** Euclidean distance of the normalized decoding values calculated between each stimulus feature. A shuffled control where stimulus labels have been shuffled prior to channel normalization and Euclidean distance calculation is shown for comparison.

We next quantified the visual difference we observed between the spatiotemporal maps for the stimulus-specific information (Figure 3). Since we were primarily interested in relative decoding performance throughout the cortical columns, we normalized each channel (electrode contact) by subtracting mean decoding performance across channels for each individual timepoint in the time series for each stimulus feature. We then calculated the Euclidean distance between each of our stimulus feature at each timepoint. These results were then compared to a shuffled label control where we similarly normalized our electrodes at each timepoint and then calculated the Euclidean distance (Figure 3B). Here, we find that the spatiotemporal differences between eye of origin, orientation, and stimulus history are all higher than the differences found in the respective shuffled label control. Eye-of-origin, which was more readily decoded in the granular layers was distinct from the decoding of stimulus orientation and repetition, which both lead to higher decoding in superficial and deeper layers. To statistically compare the differences across space and time, we next converted the searchlight matrices into one-dimensional vectors and then normalized across channels before conducting a pairwise signed rank test. Using this approach, we found significant decoding differences between eye of origin and orientation (*p* < 0.001), eye-of-origin (*p* < 0.001) and repetition (*p* < 0.001), and orientation and stimulus history (*p* < 0.001). As expected, there were no significant differences between the shuffled label controls. These decoding differences between stimulus features indicate that processing these stimulus features occurs distinctly but simultaneously with the laminar microcircuit.

### Temporal Dynamics of Stimulus Information Using Time Generalization

**Figure 4:**
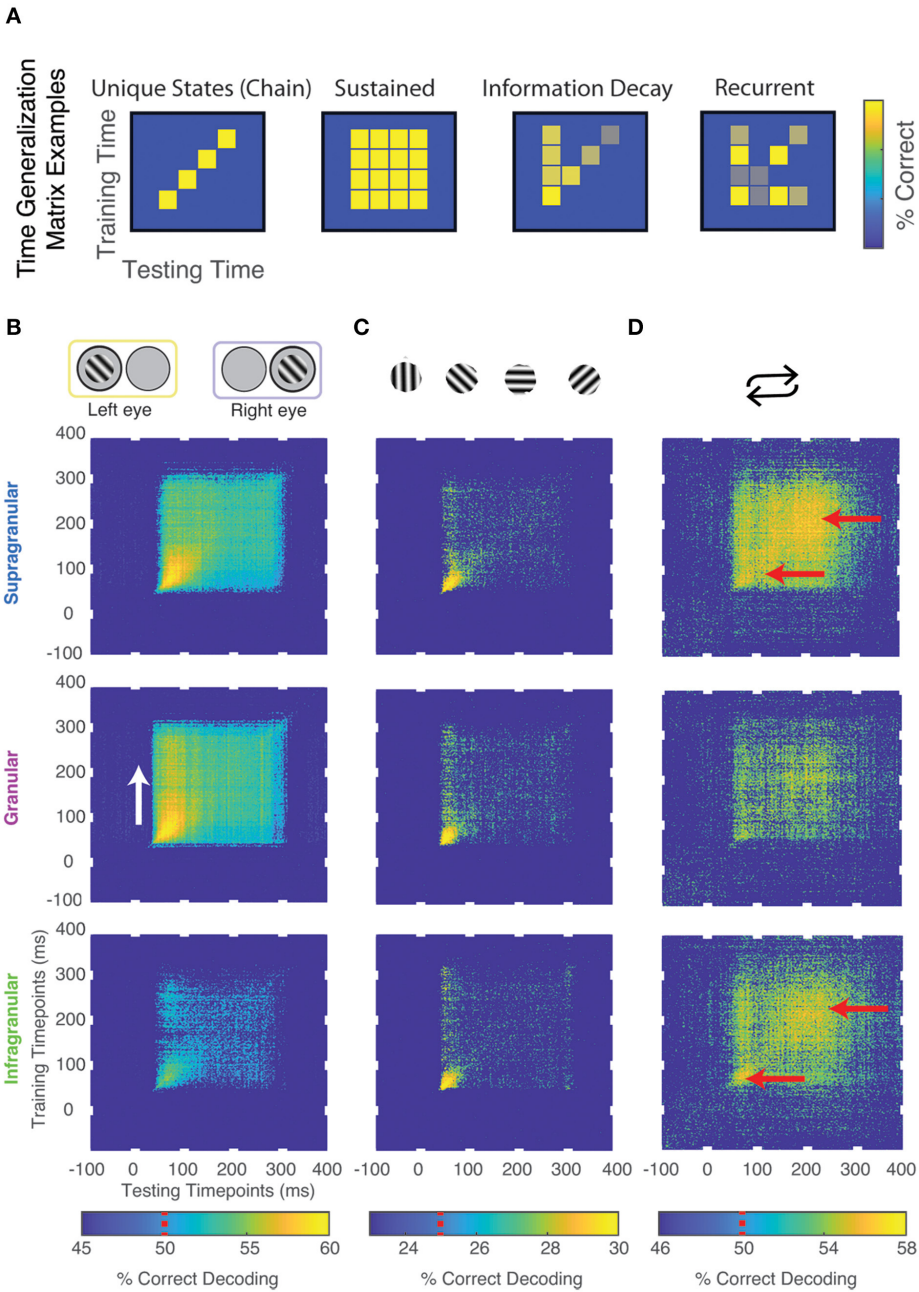
Temporal dynamics of stimulus information using time generalization. **(A)** Cartoon models of possible results. **(B)** Significant time generalization results, FDR corrected for multiple comparisons, *q* < 0.025, for: **(B)** Eye-of-origin, **(C)** Orientation, **(D)** Stimulus repetitions (see Methods for details). Chance decoding level is indicated on each color bar by a red line. Red and white arrows are added for emphasis.

To further investigate how feature information evolves over time (see also: Ringach et al., 1997, 2002, 2003; Bair et al., 2002; Smith et al., 2006; Shapley et al., 2007), we decoded neuronal data based on a classifier that was trained for another time period of the same neuronal response (“time generalization”) (Carlson et al., 2011; King and Dehaene, 2014). The result of this analysis is a 2D “time generalization matrix” that plots training time against decoding time. Figure 4A illustrates several possible outcomes for generalization matrices. It is possible, for example, that there is little to no generalization between a classifier trained at one time and tested on the remaining time of a neuronal response. In other words, spiking might be constantly changing in a way that any information used to discriminate between stimuli is specific to each individual point in time of the neuronal response (“unique states”). In contrast, if the information used to discriminate between stimuli were static across the neuronal response, we would expect a square-like pattern (“sustained”). This analysis can also show information decaying over time (“information decay”). An asymmetric pattern occurs because a classifier trained on lower signal-to-noise ratio (SNR) data generalizes better to higher SNR data than the converse (van den Hurk and Op de Beeck, 2019). Lastly, information might reoccur at a later time point of a response (“recurrence”).

We performed time generalization analysis for the decoding of eye-of-origin, stimulus orientation as well as stimulation history within each laminar compartment (Figure 3 and Supplementary Figure 2). Decoding eye-of-origin was mostly sustained but also exhibited some information decay within each laminar compartment (Figure 4). Decoding of stimulus orientation, in contrast, was less sustained. Interestingly, whether or not a stimulus preceded or succeeded other stimuli showed a very different pattern. Specifically, the time generalization matrix was suggestive of recurrent processing, in that the initial information emerges, weakens and then re-emerges at a later time point. This reactivation pattern was most prominent in the supragranular and infragranular layers (Figure 4).

**Figure 5:**
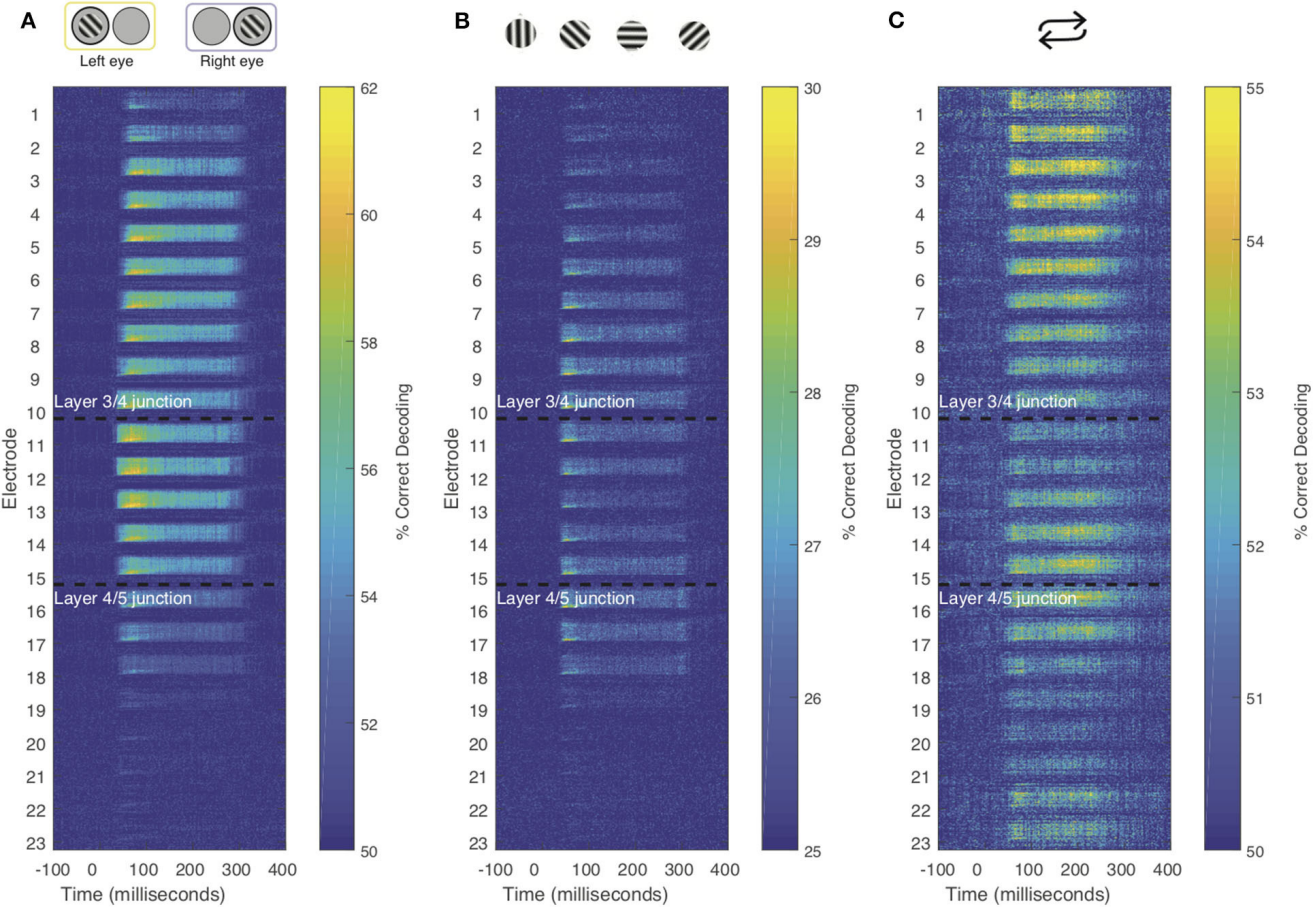
Combined time generalization and moving searchlight analysis along the depth of the linear electrode array. We performed this analysis for each of the main stimulus features analyzed in this paper: Stimulus **(A)** eye-of-origin, **(B)** orientation and **(C)** repetition. Each sub-panel shows a series of time generalization plots ranging from 100 ms before stimulus to presentation to 400 ms post stimulus presentation using a moving searchlight of three electrodes and two electrodes at the end of the electrode array.

To further investigate how the temporal dynamics for each of the stimulus features varies within compartments. We combined the searchlight and time generalization analyses (Figure 5 and Supplementary Video 1). Using this approach, we found that the electrode-specific time generalization matrices were generally representative of their respective compartments. However, within compartments there was notable heterogeneity. For example, for eye of origin decoding, time generalization was comparable across contiguous electrodes. In contrast, for decoding stimulus history (repetition), the reactivation pattern noted in Figure 5 waxes and wanes even within laminar compartments. These results provide evidence for the notion that sub-layers within laminar compartments differentially process distinct stimulus features.

## Discussion

Recent studies using linear multielectrode arrays in V1 have successfully contrasted externally evoked feedforward activation with internally generated feedback (Spaak et al., 2012; Maier, 2013; Van Kerkoerle et al., 2014, 2017). These results are encouraging as they demonstrate that the flow of neural activation across cortical layers is highly informative regarding the context of neuronal activation – an important insight that is largely absent in single electrode recordings. In this study we went beyond these earlier findings by showing how the build-up of cortical laminar activation contains several parallel streams for information specific to stimulus features that are difficult to trace using univariate analyses, even when laminar data has been obtained.

### Drawing Insight From Multivariate Spiking Profiles

In recent work, layer-specific processes are often grouped to perform univariate analyses to investigate differences between layers (see Westerberg et al., 2019 for example). This is because we often consider cortical processes that follow a model known as the canonical cortical microcircuit (see Bastos et al., 2012 for review). This model hypothesizes three functional compartments in granular cortex: a feedforward recipient granular compartment sandwiched between supragranular and infragranular compartments. While this model has provided powerful insight into cortical function, we know that even within layers there can be degree of heterogeneity in the distribution of neurons. That is, neuron “A” might exist in layer 2 of cortex where neuron “B” exists in layer 3. While both neurons are present in the supragranular compartment and their activity might reflect the same process, the information they carry might vary in meaningful ways. MVPA incorporates information across all channels comprising a predefined laminar compartment. This allows a more integrative approach in evaluating the activity of laminar compartments than previous approaches. Namely, previous work considers independent channels from a laminar compartment representative of the compartment's overall activation state (Westerberg et al., 2019). However, information might be encoded in the dynamics within a layer that would be lost in univariate analyses.

Another advancement afforded by the MVPA approach is by being able to generalize information states across time. The time generalization analysis allows us to track patterns of information encoding. That is, by evaluating decode performance by training and testing the classifier at different time periods, we can observe how information processing is remaining consistent or evolving. A stable representation of a feature will not only be decodable at the timepoint in which a classifier is trained, but also at later timepoints. Meanwhile, with a dynamic representation, a classifier will not generalize far beyond the trained time (Carlson et al., 2011; King and Dehaene, 2014; Mohsenzadeh et al., 2018). Furthermore, we can infer how certain stimuli features vary in time and match potential models of neural encoding found across a number of studies (for review see King and Dehaene, 2014).

### Implications for the Circuitry of Binocular Combination, Orientation Representation, and Repetition Suppression

The analyses performed here further our understanding of several processes along the V1 laminar microcircuit. First to consider is the laminar profile of binocular combination. Through our analyses, we found that visual signals of each eye are more strongly integrated once they reach the deep layers. We found a drastic reduction in eye-specific information in the lower layers of V1, suggesting the information regarding eye-of-origin are largely resolved prior to the lower layers. This pattern is in line with earlier reports, locating the bulk of V1 binocular neurons in both the upper and lower layers (Hubel and Wiesel, 1977). This apparent paradox might be explained by a recent finding that a large fraction of monocular V1 neurons are sensitive to both eyes (Dougherty et al., 2019a). Thus, a neuron's preference for one or the other eye may not necessarily be predictive of how it responds to binocular stimulation (see also Read and Cumming, 2004). Furthermore, eye-specific information also seemed to decrease in both the searchlight decoding and time generalization results, indicating that it is more readily dispensed by V1's CCM compared to other types of stimulus information, which seems in line with the fact that eye-of-origin information is of low behavioral relevance (Blake and Cormack, 1979; Solomon and Morgan, 1999; Schwartzkopf et al., 2010). While our findings regarding the representation of eye information the lower layers requires more direct testing to reconcile with previous work, our other finding that each eye's stream of information stays largely separate until visual activation reaches the upper layers of V1 are compatible with hypotheses regarding the origins of binocular combination.

Our results also revealed a fine-grained spatiotemporal laminar pattern of orientation tuning, with some but not all sublayers of granular layer 4 exhibiting less sensitivity to stimulus orientation than the superficial and deep layers of V1. Although it is not immediately clear how the specific pattern produced by MVPA relates to the magno- and parvocellular recipient sublayers, our finding seems to be generally in line with the idea that V1 receives at least some LGN inputs that are somewhat “biased” toward certain stimulus orientations, with further processing within V1 producing the more discerning orientation tuning that characterizes this area.

With respect to the circuitry of adaptation in V1, it is interesting to note that stimulus repetition yielded a unique signature of time generalization in the feedback-recipient layers of V1. Previous work suggested that adaptive changes largely arise from changes in feedback activation in V1 (Westerberg et al., 2019). The temporal features of this time generalization pattern are somewhat reminiscent of prior descriptions of feedback modulation in V1 (Van Kerkoerle et al., 2014). However, our finding goes beyond the demonstration of a secondary peak in activation by revealing that the information content within this activation is specific to contextual information.

### Sources for Feature-Specific Activation Patterns in V1

It is interesting to speculate as to the source of these differences in layer-specific information flow. Could it be that differences arise through differences in processing local to V1 or is another brain area affecting feature-specific change in the V1 laminar microcircuit? Previous work has begun to investigate such questions. For example, investigation into the origins of adaptation resulting from visual repetition suggests that the reduction in neural responses in V1 associated with visual repetition comes about through a reduction in the feedback activity to the V1 laminar microcircuit rather than through changes in feedforward processing local to V1 (Westerberg et al., 2019). This is in contrast to the process of binocular combination which is largely thought to be accomplished even prior to the feedforward activation of the supragranular layers of V1. It is through these differences in activation that might elicit the observed differences in information flow along the layers. Further investigation, perhaps through causal inactivation of feedback connections to V1 (Nurminen et al., 2018), would shed light on whether feedback activation is indeed necessary for the observed patterns of information flow described here.

### Toward Ultra-High-Resolution Laminar Neurophysiology

We are on the cusp of a revolution in primate neurophysiology that will allow for massively increased insights into the function of mesoscopic neural circuits (Jun et al., 2017; Steinmetz et al., 2018; Musk and Neuralink, 2019). Modern recording technologies have advanced to allow for the simultaneous recording from thousands of channels. This substantial advance in resolution of data allows for the interrogation of data through novel analytical methods. With increased resolution of data comes the ability to investigate data in more integrative approaches. MVPA has proven highly useful in the functional imaging literature where large multichannel datasets have been commonplace for decades. Through the analyses demonstrated here, we propose these same analyses as useful approaches to investigating ultra-high-resolution neurophysiology as these recording techniques become more and more common.

## Data Availability Statement

The data analyzed in this study is subject to the following licenses/restrictions: None apply. Requests to access these datasets should be directed to alex.maier@vanderbilt.edu.

## Ethics Statement

The animal study was reviewed and approved by Vanderbilt IACUC.

## Author's Note

An earlier version of this this manuscript has been released as a pre-print at bioRxiv (Tovar et al., 2019).

## Author Contributions

DT, JW, and AM conceptualized the study. TC, MW, and AM supervised the study. JW, MC, and KD collected the data. JW preprocessed the data. DT performed data analyses and created figures. DT, JW, and AM wrote the initial draft of the paper. DT, JW, MC, KD, TC, MW, and AM revised the paper and approved the final version of the manuscript. All authors contributed to the article and approved the submitted version.

## Conflict of Interest

The authors declare that the research was conducted in the absence of any commercial or financial relationships that could be construed as a potential conflict of interest.
