# Supplementary material for: Stimulus Feature-Specific Information Flow Along the Columnar Cortical Microcircuit Revealed by Multivariate Laminar Spiking Analysis
Source: Front Syst Neurosci. 2020 Nov 30;14:600601. doi: 10.3389/fnsys.2020.600601 (PMC7734135; doi:10.3389/fnsys.2020.600601)
Supplement: Supplementary file 2 [file Data_Sheet_1.PDF]

Supplementary Material

1 SUPPLEMENTARY FIGURES

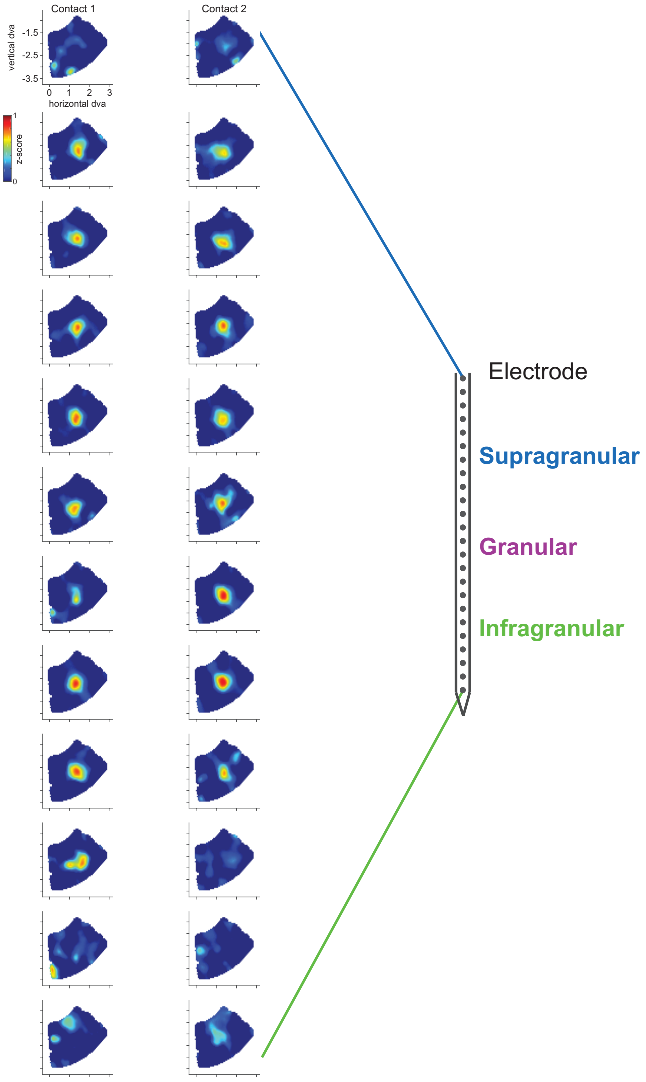

Figure S1. Receptive field mapping. For each contact of the linear multielectrode array, we computed the magnitude of MUA spiking responses as a function of visual field stimulation using a reverse-correlation technique (see Methods). Colored plots to the left show averaged neuronal response in units of standard deviation as a function of angle and magnitude in visual degrees. Panels are arranged in descending order with each column representing neighboring channels on the electrode array so that each row represents the electrode channel that is 200 microns below the channel above. Note that the receptive field locations deviate little between the top and the bottom of the array, indicating that the electrode was inserted perpendicularly to the cortical surface. The topmost and bottommost channels of the array produced no visual responses as these electrode channels reached outside the cortical thickness.

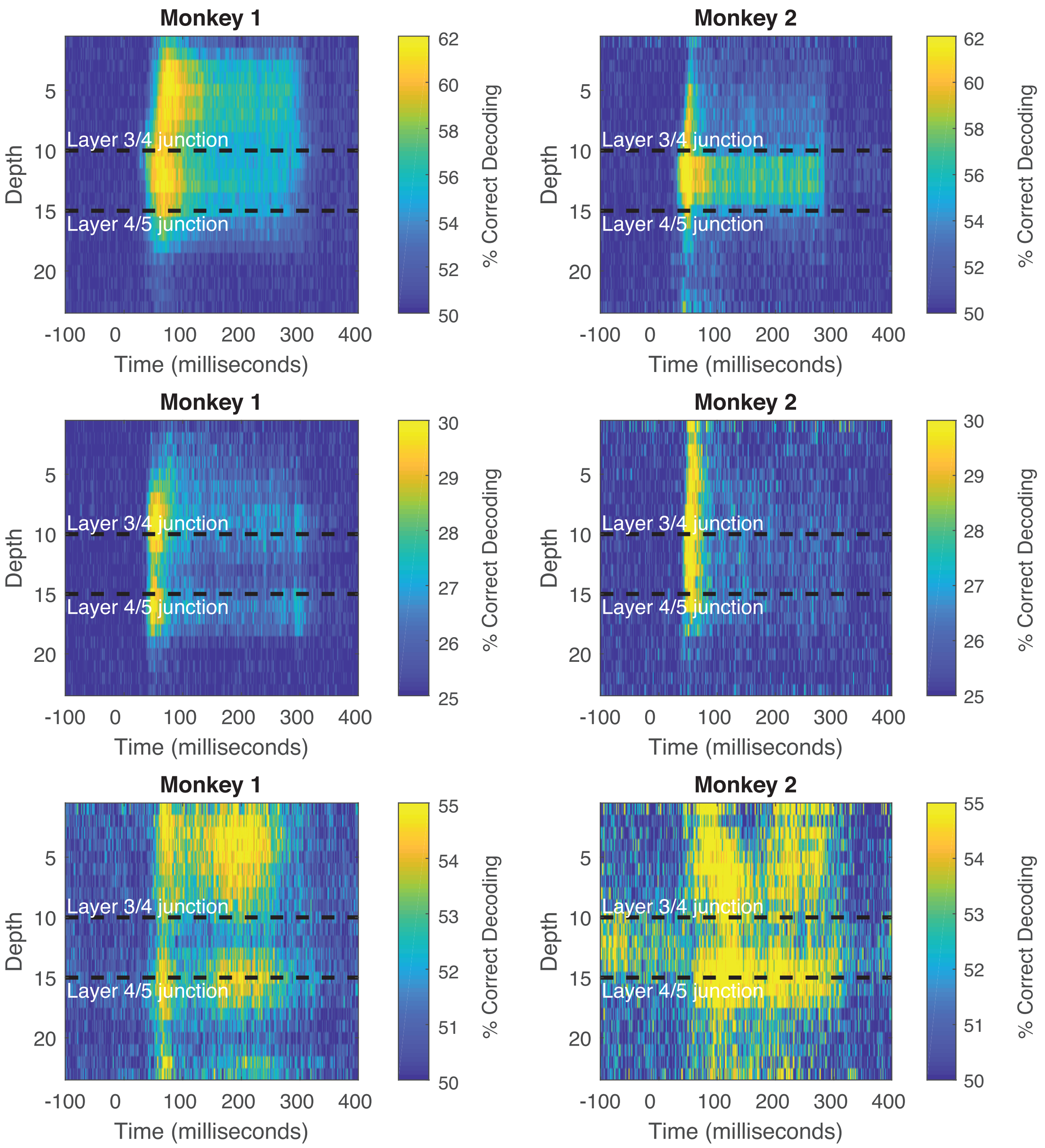

Figure S2. Searchlight analysis separated by monkey. Decoding performance using a moving searchlight along the electrode array for (A) eye of origin, (B) grating orientation, and (C) stimulus repetition. Monkey 1 (left panel) and Monkey 2 (right panel).

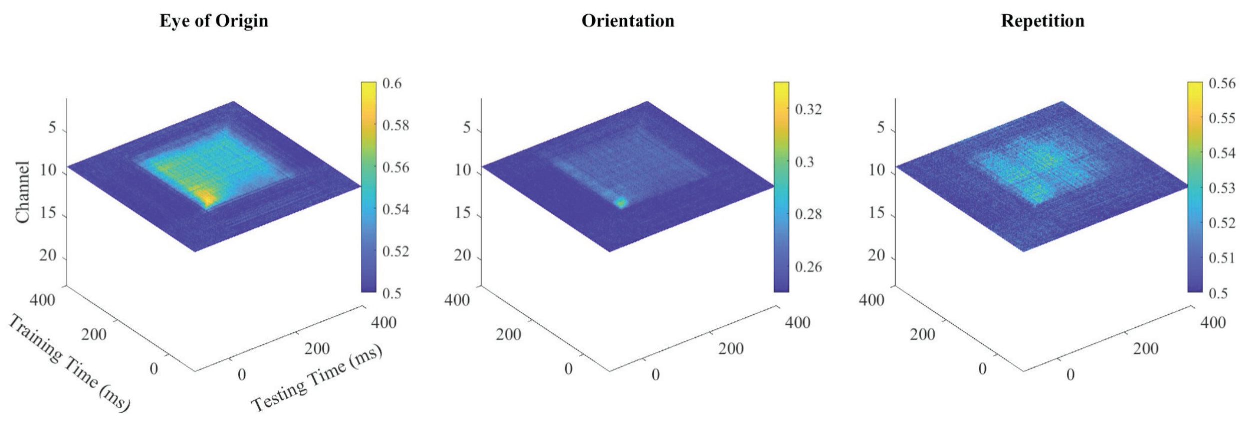

Figure S3. Video of Combined Time Generalization and Searchlight Analysis
